# Detection and Mitigation of DoS and DDoS Attacks in IoT-Based Stateful SDN: An Experimental Approach

**DOI:** 10.3390/s20030816

**Published:** 2020-02-03

**Authors:** Jesús Galeano-Brajones, Javier Carmona-Murillo, Juan F. Valenzuela-Valdés, Francisco Luna-Valero

**Affiliations:** 1 Department of Computing and Telematics Engineering, Universidad de Extremadura, 06800 Mérida, Spain; jcarmur@unex.es; 2 Department of Signal Theory, Telematics and Communications, Universidad de Granada, 18071 Granada, Spain; juanvalenzuela@ugr.es; 3 ITIS Software, Universidad de Málaga, 29071 Málaga, Spain; flv@lcc.uma.es; 4 Department of Languages and Computer Science, Universidad de Málaga, 29071 Málaga, Spain

**Keywords:** stateful SDN, DoS, DDoS, entropy, Internet of Things, experimental evaluation

## Abstract

The expected advent of the Internet of Things (IoT) has triggered a large demand of embedded devices, which envisions the autonomous interaction of sensors and actuators while offering all sort of smart services. However, these IoT devices are limited in computation, storage, and network capacity, which makes them easy to hack and compromise. To achieve secure development of IoT, it is necessary to engineer scalable security solutions optimized for the IoT ecosystem. To this end, Software Defined Networking (SDN) is a promising paradigm that serves as a pillar in the fifth generation of mobile systems (5G) that could help to detect and mitigate Denial of Service (DoS) and Distributed DoS (DDoS) threats. In this work, we propose to experimentally evaluate an entropy-based solution to detect and mitigate DoS and DDoS attacks in IoT scenarios using a stateful SDN data plane. The obtained results demonstrate for the first time the effectiveness of this technique targeting real IoT data traffic.

## 1. Introduction

In recent years, we have witnessed a popularization of communications networks, which has allowed users to be connected at any time and almost anywhere, thus generating growing traffic demand. The proliferation of different smart devices and applications, as well as the development of a wide range of network technologies, are generating an unprecedented amount of data traffic. Thus, the expected traffic growth in the global Internet in 2019 will exceed 200 Exabytes per month, reaching 396 Exabytes per month in 2022 [1].

In addition, the rise in the number of objects connected to the Internet has made Internet of Things (IoT) an increasingly growing topic in recent years and it is expected that it will exponentially increase in the coming years. Several forecasts project that the current number of connected devices at the end of 2019, around 1.3 billion, will reach 5 billion IoT devices in 2025 [2]. In this context, the explosive rise of IoT is leading to the creation of new advanced services with more stringent requirements such as low response time and low energy consumption. Services such as the industrial IoT, automotive IoT or e-health are typical IoT-enabled critical infrastructures that require the network to be ready to provide the proper network capabilities and also to cope with different security challenges [3].

Indeed, to achieve the success of IoT, it is necessary to develop advanced mechanisms able to ensure proper security levels to detect cyber-attacks and mitigate cyber-threats whenever occur in the managed IoT network. This poses a great challenge as IoT devices may handle sensitive information and many commercial IoT low-end devices do not usually support strong security mechanisms, making them easy targets to conform the malicious network of devices for different attacks such as DoS (Denial of Service) and DDoS (Distributed Denial of Service) [4].

Moreover, new emerging technologies such as Software-Defined Networking (SDN) and Network Function Virtualization (NFV) are a major pillar in the incoming fifth generation of mobile networks (5G) [5,6]. These technologies will make the network more flexible as a new functionality can be introduced with simple software upgrades, and more sophisticated algorithms can be employed to manage the network. Traditional networks often involve the integration and interconnection of many proprietary and vertically integrated devices which, in addition to proprietary software and closed development, makes it extremely difficult to introduce and deploy new protocols in the network [7]. SDN is a new network paradigm proposed to change the limitations of current network infrastructures, breaking the vertical integration by separating the network’s control logic (the control plane) from the underlying routers and switches that forward the traffic (the data plane), which gives more flexibility to the network. Also, because of this separation of planes, network switches become simple forwarding devices and the control logic is implemented in a logically centralized controller, simplifying policy enforcement and network (re)configuration and evolution. Additionally, both control and data planes in these devices are integrated into most commercial networking devices, which makes IP networks difficult to manage [8]. The appearance of SDN is moving towards open software and network hardware, allowing an easier mechanism for developing and testing new solutions and protocols in production environments. The major principle in the SDN paradigm is the decoupling of the control plane, based on software, from the data plane, which is based on hardware. Thus, the logic of the network, or control plane, resides in a central controller that manages the network according to the different applications running on top of it (application plane). For its part, the packet forwarding (data plane) is done in the network equipment, which steers traffic according to a set of rules created by the SDN controller.

NFV is an evolving network approach that allows the replacement of expensive, dedicated and proprietary hardware (such as routers, firewalls, etc.) with software-based network devices, by decoupling network functions from the underlying hardware. NFV also allows instances of virtual functions to be shared by several clients [9]. NFV proposes that the functions performed by the network nodes are defined in a virtual model, so that the network services functions can be classified within blocks that can be chained. Each of these blocks would represent a Virtualized Network Function (VNF). These functions provide specific network functionalities, such as encryption/decryption, VPN, load balancing, firewall, etc. Service virtualization provides faster deployments, reducing OPEX (OPerational EXpenditure), energy consumption and improving adaptation to new business models.

Although SDN and NFV have achieved great success, recent research in these technologies reveals potential security challenges that must be addressed to ensure the required security of new 5G services and infrastructures [10]. In this 5G environment, the massive use and growing expectation of IoT technology requires sophisticated mechanisms that are able to detect and mitigate the threats that IoT devices and smart objects may be exposed. In general, the goal of a vast majority of the attacks is to limit or deny the services through an increasing variety of DoS and DDoS attacks [11,12].

This paper presents a stateful SDN security solution for real IoT traffic that can detect and mitigate DoS and DDoS attacks based on the concept of entropy as the detection method, whose advantages are the high sensitivity, low false positive rate for correct adjustment of algorithm parameters, the simple calculating of the entropy and no need of additional network device. The key point here is the stateful approach, as SDN traditionally takes advantage of OpenFlow as an abstraction of the data plane [13], which thanks to flow tables (configured by SDN applications or the controller), delegates any intelligence or control logic with states directly to the controller. Consequently, OpenFlow switches are limited to install and comply with the rules for forwarding stateless packets received from the network control plane, which generates signaling overhead and increases the latency. For this reason, several abstractions have been proposed in recent years [14] at the data plane level to offer some stateful logic to the switches, avoiding overloading the control plane and the latency increase. Thus, to the best of our knowledge, this paper shows for the first time that entropy-based mechanisms can work efficiently with real IoT traffic in stateful SDN network architectures.

We have designed three different experimental scenarios in which DoS and DDoS attacks are conducted using real data traffic from two data sets called “BigFlows” and “Bot-IoT”. In the first scenario, we have taken real traffic from the “BigFlows” trace [15]. The other two experimental scenarios have been carried out using the data set called Bot-IoT [16]. Bot-IoT has been selected because it was recently designed and its creation was based on a well-structured data set for network forensic analytics in IoT. It also incorporates legitimate IoT network traffic. Moreover, some relevant previous work in the literature dealing with (D)DoS attacks in SDN stateful architectures, such as that in Reference [17], use simple topologies to obtain the results. In the last two network scenarios presented in this work, we designed a more complex network topology in order to further investigate the performance of the solution in a more realistic scenario.

As we will mention in Section 3, several solutions against DoS and DDoS attacks based on statistical methods have been proposed in the literature. There are also different works that investigate the detection of these attacks using the stateless SDN paradigm [18,19]. However, statistical solutions in SDN architectures exhibit several drawbacks that are required to be addressed in order to achieve efficient methods for the detection and mitigation of these security hazards. On one hand, statistical solutions such as the entropy-based mechanisms are known for the detection capabilities but lacking the mitigation functions. On the other hand, the SDN paradigm shows relevant limitations to perform the detection and mitigation of the attacks due to the stateless abstraction of the data plane that generates overload and complexity.

Thus, as mentioned in Reference [20], it is critical for a statistical (D)DoS solution to have both the capacity of detection and mitigation as well as to bring reasonable computational complexity in SDN network architectures. In this context, the key contributions in our work can be summarized as follows:

- We have developed an entropy-based solution that works properly in a stateful SDN architecture. The joint utilization of the statistical solution and the stateful SDN paradigm allows the offering of a holistic proposal that both detects and mitigates DoS and DDoS attacks.
- The use of real IoT traffic in the experimental scenarios. Most of the works available in the literature deal with artificial traffic or are not IoT network traffic. As stated in Reference [20], an important issue for any defence scheme based on statistical methods is the need to work with specific traffic profiles. In this work, we have conducted our experiments with real IoT traffic.
- Design of more complex topologies for the experimental scenarios. Previous works that deal with DoS or DDoS attacks in stateless SDN networks obtain the results using simple scenarios. In order to achieve more effective results, we have designed topologies with several switches in the SDN architecture.

The rest of the article is organized as follows: the next section provides an overview of the related technologies involved in this work. Section 3 focuses on the mechanisms proposed for the detection and mitigation of (D)DoS attacks. Section 4 details the set up and the experimental evaluation conducted to obtain the results. Finally, Section 5 concludes our article and provides future directions for research.

## 2. State of the Art

In this section, we start describing the SDN architecture, as well as the different security threats that can be found in this type of architecture, highlighting which parts of the SDN architecture may be vulnerable. Next, the related work about the detection of DoS and DDoS attacks in SDN networks is shown. Finally, the entropy concept is described to show how this mechanism can be used for network security threat detection.

### 2.1. SDN

**Figure 1:**
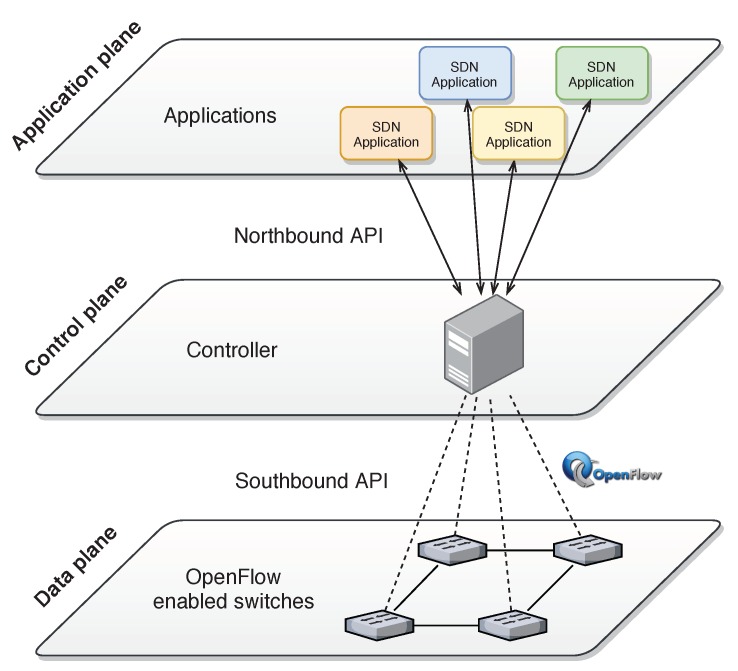
Software Defined Network (SDN) architecture.

As shown in Figure 1, the SDN controller is the key component of the control plane, as the “brain” of the network and offers interfaces to the other planes. The interaction with the application plane is made through the northbound interface, a set of open APIs (Application Programming Interface) that simplifies the process of creating network applications. The interaction with the data plane is through the southbound interface that enables communication between the SDN controller and the switches. The most common protocol used in the southbound interface is OpenFlow, an important protocol in the SDN scope, maintained by the Open Networking Foundation and supported by all major network equipment vendors.

Recently, a new approach called Stateful SDN has extended the main functionalities of OpenFlow and include the capabilities to apply different match-actions rules based on different states found in the SDN flow tables of the switch [17]. With this functionality, the switch implements the ability to react to packet-level events and free the controller and the control plane to make functions which now are made by it. If the result of the analysis matches with the rules that the switch has in the flow tables, it can act according to the rule.

SDN has also facilitated the deployment of services based on new technologies like IoT due to the decoupling of the management and forwarding plane. The development of new applications and services is optimised as the control of the network is fully distributed, and adding a new feature is simplified by deploying new applications in the controller [21,22].

### 2.2. Security Attacks in SDN Networks

The SDN architecture is composed by many types of components (Network Entities (NE), SDN controllers, SDN applications, northbound and southbound interfaces), while legacy networks were only composed by one (network devices). Now, not only must network devices be protected, but also controllers, applications and the communications between them. Table 1 summarizes the main attacks to which SDN is considered vulnerable and the respective security property involved.

The northbound interface can be defined as an attack vector because there are many APIs that are used by SDN Applications to the services implemented in the SDN controller. Northbound APIs could use different technologies and programming languages. When a malicious user attacks the vulnerabilities of such technologies or programming languages, the user can take control of the SDN network by means of the controller. In other cases, if a vulnerability of northbound API is exploited, then the attacker might be able to create their own SDN policies and thus gain control of the SDN environment. The southbound interface can be also an attack vector due to there are numerous southbound APIs and protocols used for the controller to communicate with the network elements. The main APIs are OpenFlow, Simple Network Management Protocol (SNMP); Secure Shell (SSH); or Border Gateway Protocol (BGP), among others. Each of these protocols has their methods of securing the communications to network elements, but if a malicious user attacks their vulnerabilities, the flows can be modified or created into the device’s flow-table, allowing illegal traffic or changing routes to execute a Man in the Middle attack (MITM) for example.

### 2.3. DoS and DDoS Detection Background

DoS and DDoS are two of the most difficult attacks to be mitigated due to the multiple connections request for a period of time that forces the target to slow down, crash or shut down. In IoT environments, it may even cause a long-term memory depletion of the relaying nodes due to their limited resources [23,24]. With the appearance of SDN and its ability to develop applications which can be used to detect and mitigate (D)DoS more easily than using traditional hardware, various mechanisms have been developed to improve the security of the networks [25].

The first kind of mechanisms are based on statistical/policy defence. This defence involves collecting and analyzing data samples of the network to identify malicious traffic. Various statistical algorithms are developed using different measurements like standard deviation, adaptive correlation analysis, entropy or calculate the chi-square statistic of the sample in order to classify the flow as an attack or a legitimate one. The defence is based on the policy installed in the switch, which defines the flows that are allowed to be forwarded and the other ones are defined as malicious [26].

The second one corresponds to the mechanisms based on Machine Learning. In recent years, these approaches are gaining the attention of the research community due to the application of traditional machine learning algorithms to analyze and classify different flows in order to obtain a malicious (D)DoS attack, which are obtaining good results [27].

Finally, the last one refers to the specific applications used to detect and mitigate (D)DoS attacks [28]. These specific applications are based on different research topics like blockchain to advise to other users about a (D)DoS attack, NFV to deploy services to improve the functionality of the network elements in order to detect and mitigate (D)DoS attacks, or develop honeypots using the network to mitigate (D)DoS.

### 2.4. Entropy-Based Detection

As stated previously, (D)DoS detection can rely on statistical mechanisms. Some information theory-based metrics are particularly popular in the detection of (D)DoS attacks. In information theory, Shannon’s entropy [29] is a measure of uncertainty associated with a random variable and it is assumed as one of the most effective methods for detecting abnormal traffic [30,31].

Considering that (D)DoS attacks cause significant changes in network traffic distributions, the entropy parameter can capture such variations and trigger a warning about the traffic behaviour. That is, analyzing the pattern of a (D)DoS attack, when it comes out, the fake data packets threatening a target will usually be created with different source IP addresses and randomly distributed. Thus, after computing the entropy of a parameter such as the source IP address in a series of continuous packets, a high value of the entropy means that the parameter is more random. On the contrary, a smaller value of the entropy implies a statistically high incidence for a source IP appearance. Although the entropy is a value that fluctuates between a relatively short interval, a significant change in the randomness of consecutive traffic parameters causes a high variation in the entropy values, which makes this statistical value a relevant parameter to detect some attacks.

Regarding its formal definition, the entropy is formulated as follows. Let an information source have *n* independent symbols identified as *i*, each with a probability of choice $p_{i}$. Then, the entropy *H* is defined as

(1) $$H=-\sum_{i=1}^{n}p_{i}\log_{2}p_{i}.$$

The next section describes in detail the solution developed in an SDN network to detect (D)DoS attacks through a mechanism based on the concept of entropy.

## 3. Entropy-Based (D)DoS Attacks Detection and Mitigation in Stateful SDNs

In this section a new approach to detect and mitigate the DoS and DDoS attacks based in the entropy calculation is described. To develop a model which protects the network from (D)DoS attacks, there are three stages that must be implemented—Traffic/flow monitoring; Anomaly Detection; Mitigation.

Most research implements these stages in a traditional way, in which the switches of the network only forward packets and all the control logic is implemented in the controller. This implies that, for each attacker, the switch has to send a packet to the controller to find out what action to take by adding a new flow rule. In addition, if the detection mechanism corresponds to an entropy-based algorithm, all packets must be monitored [18], so stateless solutions are not efficient or scalable.

Recently, with the appearance of the aforementioned OpenState, SDN networks are able to provide the switches with stateful packet processing capabilities [17]. That is, when a packet arrives, it will not be processed solely according to the values contained in its headers, but instead the switch will also take into account the packets that were previously received. Thus, this approach allows delegating the supervision and the detection of the malicious flows to the switches. In this work, an implementation of Openstate is used, carrying out a semi-supervision with the controller. With this approach, the switches supervise the flows but the detection of the malicious flows is done in the controller.

### 3.1. Monitoring

As mentioned before, the first stage is monitoring the network to acquire the information about the network, feeding the detection algorithm based on an entropy calculation algorithm. In this work, the information analyzed are source/destination IP, source/destination port and information about the used protocol (TCP, UDP, ICMP). In SDN, there are multiple ways to develop the algorithm to monitor the network traffic. OpenFlow integrates native monitoring using the implemented messages that define those which must be used in the communication between the controllers and the switches. The controller can obtain the flow tables of the switches, but this information is not suitable to obtain many required characteristic of the traffic, which is necessary to detect DoS attacks like Source/Destination IP.

Other approaches are based on state monitoring (OpenState). This monitoring is done only by the switch, analyzing the information independently of the controller. To do that, the switch uses its flow and state tables. This approach allows obtaining the required information to detect a (D)DoS attack because the switch can count the exact number of events for a traffic characteristic monitored, increasing the precision of the algorithm.

### 3.2. Detection

The detection phase is done by the implementation of the entropy algorithm, which analyzes the data obtained in the monitoring phase, and can detect malicious flows. Compared with other tools based on patterns [32], statistical algorithms like the entropy calculation do not require a big amount of memory or storage. This transforms these approaches in a suitable solution for its inclusion into the SDN network elements.

In Equation (2), the entropy calculation for the detection system is shown, which is based on Equation (1), where $p(x_{i})$ represents the probability of the event $x_{i}$ occurring, and *n* represents the number of events.

(2) $$H\left(X\right)=-\sum_{i=1}^{n}p\left(x_{i}\right)\frac{log_{2}\;p\left(x_{i}\right)}{log_{2}\;n}.$$

A high concentrated distribution implies a low entropy value. By contrast, a low concentrated distribution implies a higher entropy value, which is the behaviour expected to detect attacks in the networks [33,34]. One of the keys in the detection is the precision of the algorithm that handles the minimum and maximum values of the entropy. To analyse the traffic, an initial learning phase is required to feed the algorithm, calculating the entropy and the limits of the distribution, defined by Equation (3). To detect the attacks, the process is repeated at regular intervals analyzing the entropy of every characteristic (source/destination IP, source/destination port). If the entropy calculation is over or under the limits defined, then the attack is detected. These limits are calculated as:

(3) limits=μ±θσ,

where μ is the mean of the previous entropy measures, θ the category and σ the standard deviation. The limits give us the certainty that a threat is occurring according to Table 2.

### 3.3. Mitigation

Finally, the mitigation mechanism protects end-users when a malicious attack is detected. To mitigate the attack, new flow rules are added to the switches with high priority to match the rule with the suspicious package. The precision of the mitigation rules depends on the amount of information of the attack can be acquired. The controller configures the rules in the switches using OpenFlow standard messages, sending messages FlowMod. There are different mechanisms to mitigate the attack like black holes, the Intrusion Detection System (IDS), even advanced techniques such as Deep Packet Inspection (DPI). In this work, the mitigation algorithm is implemented, configuring different rules in the switch, which drops the suspicious packets.

**Figure 2:**
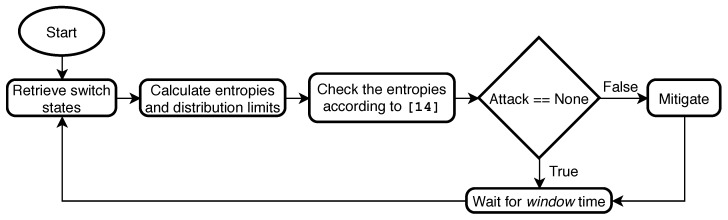
Algorithm flowchart.

In Figure 2, the flow chart of the entropy-based algorithm is shown. First, the retrieval of the states of the data plane is performed and the entropy of the system and the limits of the distributions of each of the monitored parameters are calculated; secondly, it is checked whether the entropy values are anomalous and whether they agree with the trends described in Table 1 of [17]; then, if a threat is detected, it is mitigated by sending the corresponding flow rules to the data plane; finally, a window wait is made and the algorithm starts again.

## 4. Experimental Evaluation in the Detection and Mitigation of (D)DoS Attacks

In this section, we describe the experimental scenario used to derive the obtained results. The testbed is based on the well-known SDN-based emulator Mininet [35]. This software is widely used for reproducible network experiments, as it allows the execution of unmodified Linux applications on top of an emulated network, using the real Linux kernel network stack. The SDN controller used is Ryu [36], an open-source controller with a component-based architecture. It has a set of well-defined APIs for network application development and it has been widely used by the research community. In the experiments described next, Mininet emulates one switch running the Open Virtual Switch (OVS) with the OpenState extension that implements the Stateful SDN features over a standard OVS. Next, we describe three testbed scenarios, the first one is used to show the bandwidth consumption during the DoS attack and mitigation process, as well as the entropy values in this period. The second scenario has been used to test the behaviour of the detection and mitigation process of a DoS attack in an IoT environment. The last one has the same goal as the second but with a DDoS attack.

### 4.1. DoS Attacks Base Scenario

**Figure 3:**
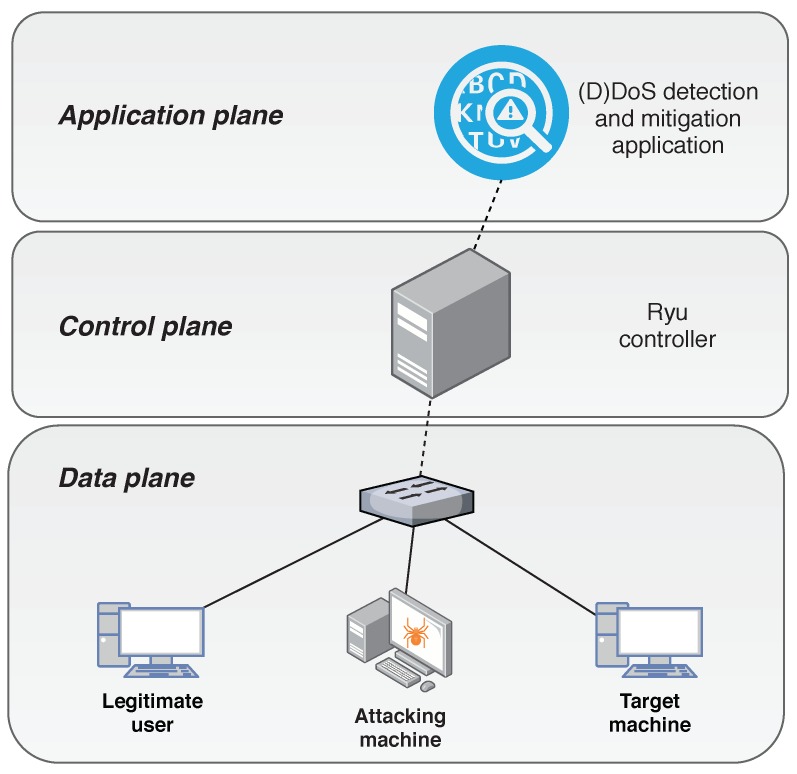
Testbed topology of the first scenario.

In this experimental setup, we have considered a network topology like the one presented in Figure 3. In this case, one Ryu controller is managing the network and the application in charge of the detection and mitigation is running at the top of it. The hosts are labelled as Host1 to Host3, and the network switch is tagged as S1. In this network, two of the hosts (H1 and H3) are regular nodes sending and receiving normal traffic whereas H2 is the attacker which will perform the DoS attack against the H3 host. In this environment, the following tests are performed. First, we conduct an experiment to check whether the detection and mitigation processes of the DoS attack are carried out correctly. In order to show the variations of the entropy under this kind of threats, the value of the entropy is measured. Additionally, the bandwidth at the switch ports is also measured to evaluate the process of mitigation. In this experiment, θ=3, window size is 3 s and the attacking machine is injecting packets every 100 µs.

The timeline of the attack is as follows. H1 is a legitimate host sending regular traffic to H3. This connection will be opened during all the experiment so, at some point, normal and malicious traffic will be mixed. The attacker H2 is connected to the switch through the s1-eth1 interface and will be responsible for generating the DoS traffic. Thus, this host launches a flooding UDP attack targeting the victim H3 sending fake UDP packets at a rate of 1 µs.

**Figure 4:**
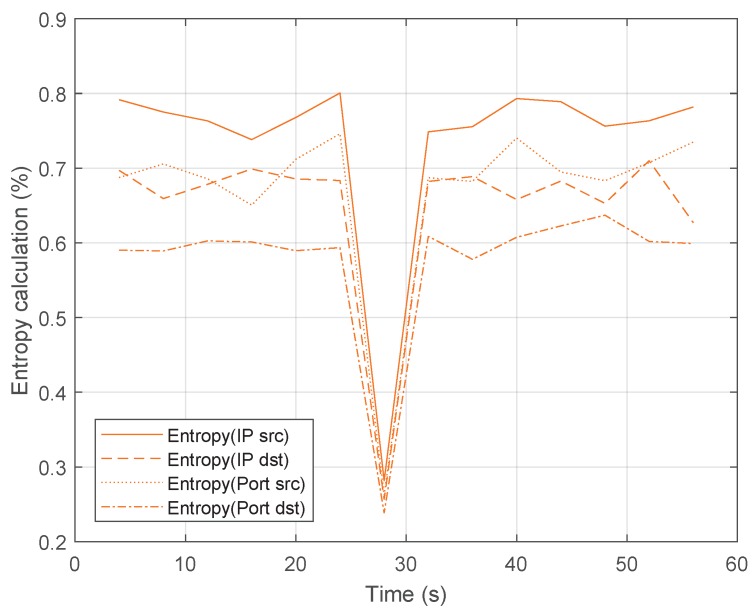
Entropy values during the Denial of Service (DoS) attack.

Under these conditions, the DoS attack starts at 26 s. Figure 4 shows the values of the entropy for some parameters such as source IP, destination IP, source port and destination port. These four features have been selected because, according to the literature, different variations in the measured entropy of these traffic features can lead to the detection of anomalies in the network traffic [17]. Before the attack, the entropy oscillates between a narrow range for each feature. Later, when the attack starts, the entropy value drops and the detection algorithm detects it. In this case, the entropy falls to very low values.

**Figure 5:**
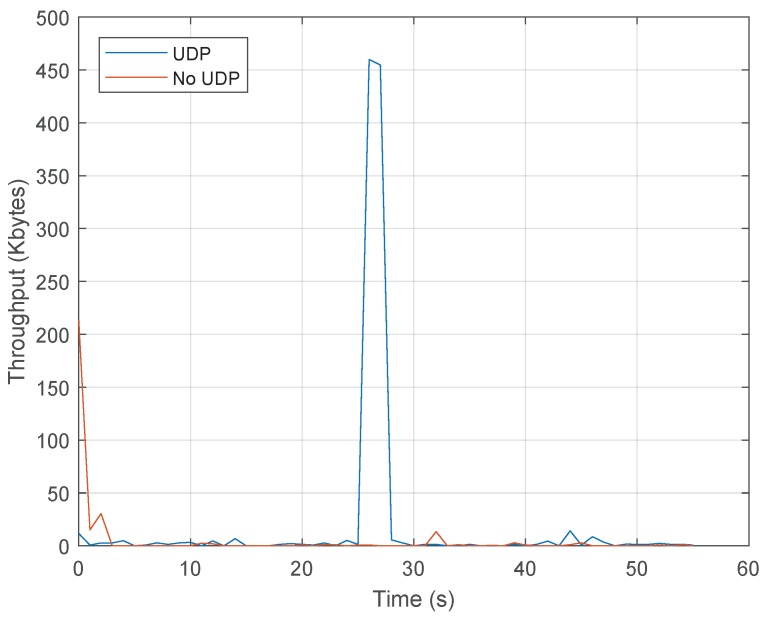
Throughput values during the DoS attack.

This result demonstrates that the anomaly caused by a UDP flooding attack can be detected by the algorithm. Next, once the attack is detected by identifying significant changes in the randomness of consecutive traffic features distribution, the mitigation process starts and, according to the application, the controller sends a message to the switch to add a new entry at the flow table that stops the attack. That is, the new flow table entry matches the packets with the source IP, destination IP, source port and destination port that are causing the attack. The instruction associated with this flow entry is to drop these packets. For example, for attacker IP 192.168.100.101 and source port 40, and target IP 192.168.100.103 with target port 2000, the flow rule contains the following values: source_IP = 192.168.100.101, src_port = 40, dst_IP = 192.168.100.103 and dst_port = 2000. This effect in the traffic flow of the attacker is represented in Figure 5. As shown, the bandwidth of the traffic generated by H2 before the attack is almost negligible and once the attack starts, the bandwidth grows sharply. Once the attack is mitigated, the bandwidth is almost zero because the switch is discarding all packets that belong to the malicious flow.

### 4.2. DoS Attack in an IoT Scenario

**Figure 6:**
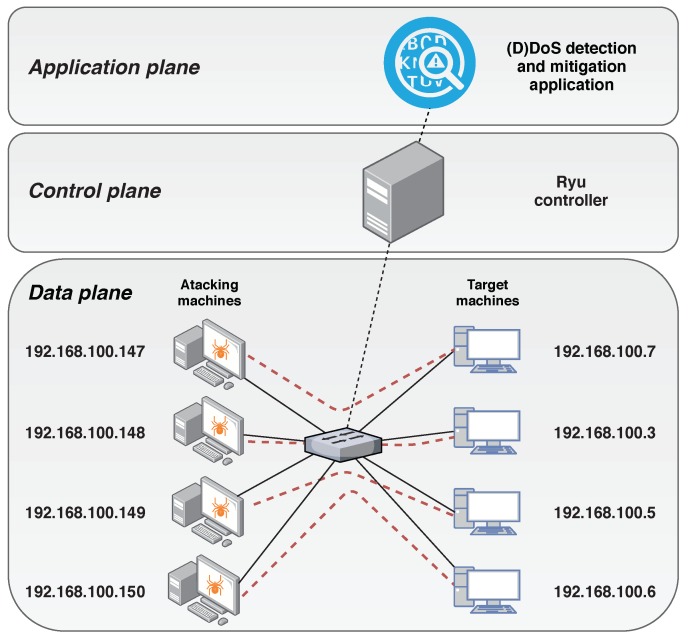
Test bed topology of the second scenario. Adapted from [16].

In order to show the effectiveness of the solution in an IoT scenario, we have conducted a second evaluation using a realistic IoT data set called Bot-IoT [16]. The topology used in this case is shown in Figure 6 and is adapted from the Bot-IoT topology used to obtain the traffic data set.

In this data set, both legitimate and malicious traffic are mixed, which makes this data set appropriate for the purpose of this work, because the results may be useful for other IoT scenarios. The IoT elements have been introduced by employing the popular Node-red middleware to simulate the existence of IoT devices in the network. The traffic generated by the IoT devices is MQTT, a common publish-subscribe protocol widely used in lightweight IoT communications. In Figure 6, the IoT simulated devices are located in the target machines, whereas the attacking hosts, performing the DoS attacks are Kali VMs. The normal traffic is sent among the target machines that, apart from the IoT traffic, are executing different services such as DNS, email, FTP, HTTP and SSH. The IoT traffic is also included in this normal traffic and a smart-home configuration has been designed to simulate the IoT network. From the attacking perspective, the DoS attacks are launched from Bots running the hping3 tool. More information about this set up is detailed in Reference [16].

**Figure 7:**
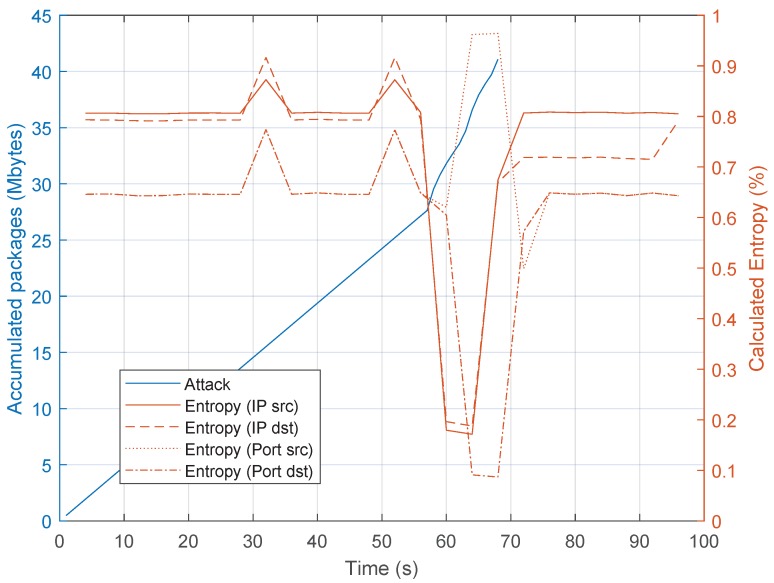
Entropy variation for a DoS flooding with spoofed src port attack in Internet of Things (IoT).

Our scenario, as shown in Figure 6, has been adapted from the setup of the Bot-IoT data set to fit into an SDN network. In this case, four DoS attacks have been launched, all of them as UDP DoS. For simplicity, and to offer a comprehensive behaviour of the parameters, we only show the results one of the attacks. The other three UDP DoS attacks have similar behaviour. Figure 7 shows the entropy of the tuple source IP, destination IP, source port and destination port. As we mentioned previously, according to the literature, by correlating the entropy variations of these four features, it is possible to identify different types of attacks.

In this case, the fluctuation of the entropy remains almost stable until second 60, when the attack is launched. In this case, the source IP, destination IP and destination port entropy fall down to values around 0.1 and source port increases to almost 1. This is a remarkable difference with respect to the entropy shown in Figure 4, in which the four values decreased. This correlation shows the kind of attack and that the application running at the SDN controller can detect that; in this case, a DoS flooding with a spoofed source port attack has been detected. After the detection, the controller sends a FlowMod message to the switch in order to add an entry in the flow table to drop all packets arriving from the attacker so, the attack can be mitigated. It is worth mentioning that the application developed monitors the switches at regular intervals of 4 s and the information collected from the switches is used for the calculations of the entropy and the kind of attack.

### 4.3. (D)DoS TCP SYN Flood Base Scenario

**Figure 8:**
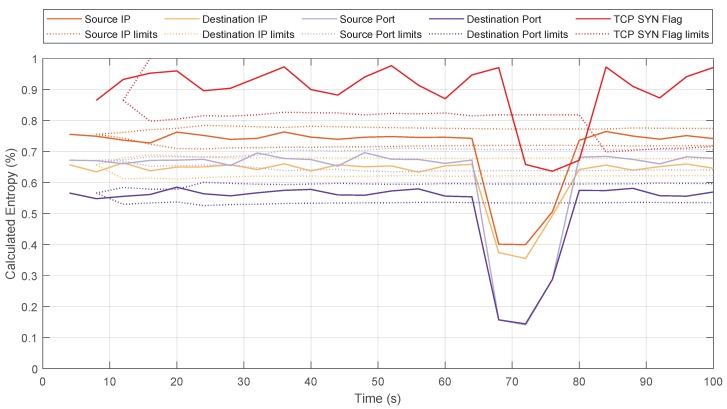
Entropy variation for a Distributed Denial of Service (DDoS) TCP SYN flood attack.

Based on the topology shown in Figure 3, but with 15 attackers instead of a single one, we performed the detection and mitigation of DDoS TCP SYN flood attacks. These attacks are characterized characterized by flooding the target with a large volume of TCP messages with the SYN flag active to establish a TCP session with the target and, since the target has to respond to these requests, the denial of service is carried out because the target cannot respond to all of them. Figure 8 shows the value of the entropy for this type of attack, considering the parameters previously used. In this case, it is also shown the entropy of the TCP packets with only the SYN flag active.

This type of attack is more complex to detect than UDP attacks, since in UDP the target does not have to respond to these messages, unlike TCP, as described previously. In these TCP threats, the detection of the attackers using entropy-based algorithms may be erroneous because the target host respond to SYN requests with a SYN-ACK message. This could lead to considering the target host as an attacker. Due to stateful SDN, we can monitor, from the data plane switches, only those TCP packets that contain the SYN flag and, when calculating its entropy, only detect the attackers since the target would not be in this state table (because it responds with SYN-ACK flags, not only the SYN flag). Therefore, the detection of the attackers is completely accurate, ensuring that the target is only detected as a victim and that the traffic belonging to the target is not affected.

**Figure 9:**
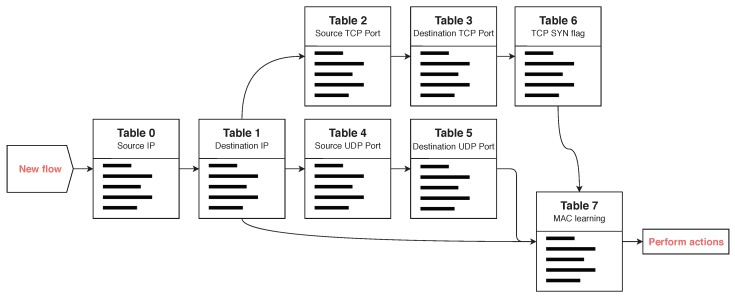
Pipeline for new flows in the state tables.

To address this point, we have added a new state table to the pipeline of new flows (table 6 in Figure 9). This state table contains a tuple [Source IP, Source Port] as key and an associated integer state; therefore, these parameters are taken from each of the flows that arrive at this table from other state tables with an initial state equal to 0. After this, the flow is forwarded to a flow table, in which it is only matched if the flow in question corresponds to a TCP packet with only the SYN flag. If this is the case, the flow returns to the state table 6 and the state for that key is increased by one unit. Thus, when the control plane requests states from the data plane, the entropy of the TCP SYN flag can be calculated and (D)DoS SYN flood attacks can be detected, detecting the IPs of the attackers, the attacking ports (in the case of a non-spoofing source port attack) and the target.

### 4.4. DDoS Attack in a IoT Scenario

**Figure 10:**
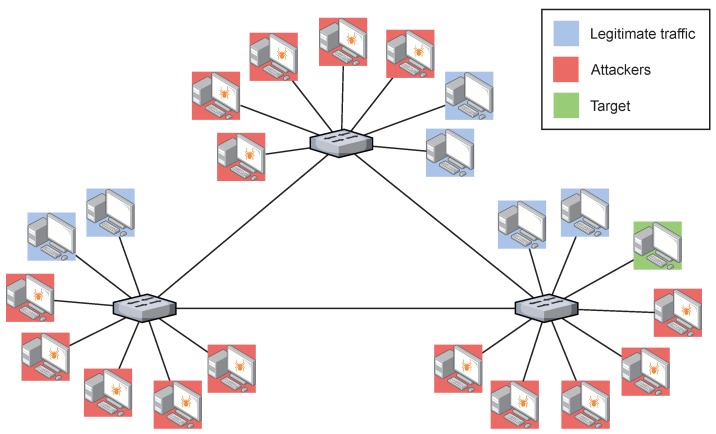
Test bed topology of the third scenario. Adapted from [16].

To conclude the experimentation and check the effectiveness of the solution with more complex attacks, we have performed a third evaluation using the Bot-IoT data set [16]. In this case, the topology used is shown in Figure 10, which is adapted from the Bot-IoT topology used to obtain the traffic data set. In this case we also add 15 attacking hosts running the hping3 tool. In Figure 10, it can be seen that the hosts with a blue background correspond to legitimate hosts (sending the legitimate traffic of the Bot-IoT data set to the target and the rest of the topology host), while the hosts that perform the DDoS attack are coloured with a red background. Finally, the target host corresponds to the green host.

The architecture of the topology is similar to the previous case, using the same control plane and application plane, but with a more complex data plane. This new data plane is made up of 3 logical switches connected in a ring, to which the different legitimate hosts, attackers and the target are connected to. In addition, the attack corresponds to UDP DDoS traffic.

First, Table 3 shows the experimentation performed for different values of the window and for θ. Finally, entropy and throughput values are shown for one of the cases.

The specifications of both the hardware and software used for this experimentation are as follows:

- Virtualization. Mininet VM 2.2.2 (Ubuntu 14.04.4) in VirtualBox with 2GB RAM and 4 CPU cores.
- Physical CPU. Intel i5-8250U.

In the experiment, 10 executions were made for each of the cases, that is, for each window size (3, 5, 10 and 20) and θ (1, 2 and 3). In addition, the algorithm had a period of 60 s in which no attack is allowed. This was done to reproduce a real situation in which the network is working properly before the attack is performed. In our case, this time is enough for the entropy calculation. Thus, the attack was launched between second 60 and 100, leaving after detection at least 3 window sizes to check if false positives were produced by the variation of the entropy values after mitigation. As a result, the detection rate, false positive rate, mean mitigation time (time elapsed since the control plane receives the states of the data plane and sends the flow rules for mitigation) and standard deviation were obtained. The detection time is not shown because it depends on the window size and the specific instant in which the attack occurred at the same time. Without loss of generality, this detection time can be considered half of the window size.

As Table 3 shows, the detection process was correctly done in most cases. Only in 3 executions was the attack not detected. This occurred when the attack began in an instant at the end of the window size, which caused the entropy values not to vary enough to reach their corresponding thresholds. In these cases, when the entropy values were not involved in an attack, those values were considered also in the next window. This means that, if the entropy was not considered to belong to a threat, those values modified the distribution limits for the next window, causing an abnormal value of the thresholds. Thus, in the next window, no attack was detected because the entropy values did not exceed the limits due to the aforementioned situation.

It can also be seen how the rise of θ generates a descent of false positives (especially from θ=1 to θ=2), so the window and θ values should be modified depending on the system traffic. The false positives rate with θ=3 is 20%. It is worth noting that this value was obtained when the highest accuracy was set to θ=3. In these cases, the algorithm needs a much longer "learning" period to correctly adjust the limits of the distributions. This is due to the fact that after an attack is detected and mitigated, the entropy varies sharply, leading to the detection of false positives. This 20% of false positives rate (indicated by * in the Table 3) always occurred after the attack was mitigated. After that, no false positives were detected, that is, for this scenario the false positive ratio was 0%. Finally, it is worth mentioning that the mean mitigation time rose if we increased the window size, as well as the standard deviation. This is due to the algorithm needing to process more states from the data plane with large window sizes, so the entropy computation time also increased.

**Figure 11:**
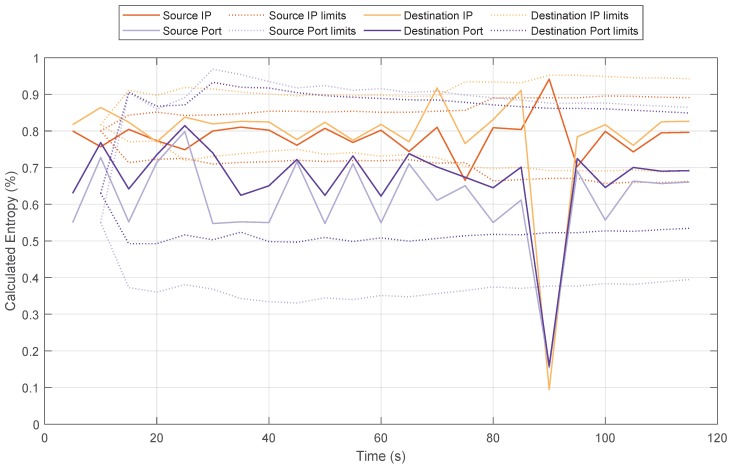
Entropy variation for a DDoS flooding attack in IoT scenario with 15 attackers.

**Figure 12:**
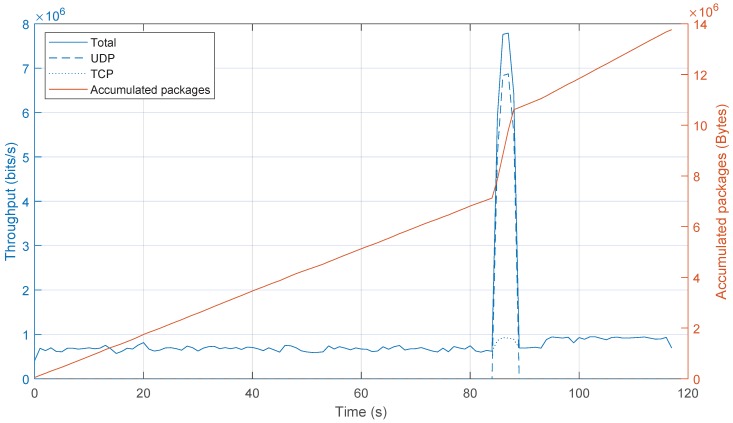
Throughput values during a DDoS attack.

Next, Figure 11 and Figure 12 show the values of the entropy and throughput respectively for the target host, with a window size of 5 s and θ=3. Figure 11, shows how the entropy values of the system varied (between seconds 85 and 90) when the DDoS attack was detected. In that period, the limits of the distribution (Equation (3)) were exceeded and the algorithm detected the attack. For the DDoS case, the source IP entropy exceeded the upper limit, and destination IP and source/destination ports exceeded the lower limit [17]. Once the attack was mitigated, the entropy returned to normal values.

Once the attack was detected, the mitigation was carried out by sending new flow rules to the corresponding switches to drop malicious traffic, keeping legitimate traffic unaltered. In Figure 12, the throughput in the target host is shown. The Figure shows the UDP packets (attacks), TCP packets (legitimate traffic) and total traffic, in addition to accumulated packets. Therefore, it can be verified how legitimate traffic is still forwarded to the target host during the attack and once the attack has been mitigated. The slight increase in legitimate traffic after the attack is due to TCP retransmissions. Figure 12 also shows how the accumulated traffic during the attack rose significantly. However, the trend is similar before and after the attack.

## 5. Conclusions

In this article, we describe a stateful SDN solution that is able to detect and mitigate DoS and DDoS attacks in IoT networks. The mechanism is based on OpenState, an extension to current OpenFlow that exploits in-switch capabilities and has been proved to be a promising approach for network monitoring since it avoids sending packets to the controller. Based on this research, we have developed a proof-of-concept application at the top of the Ryu SDN controller that detects the DoS and DDoS attacks according to the entropy values. To analyze the effect of this metric under different conditions, we have evaluated the performance of the application in three scenarios. The first one is a general test bed in which the bandwidth and the entropy values are measured during the attack, whereas the second and third focuses on an IoT scenario. The experimental results demonstrated the benefits of using the correlation of the entropy values of different features to detect the attack and also the ability of SDN to mitigate easily just adding entries to the flow table of the switches.

In the future, this work will be extended to generalize other types of (D)DoS attacks and also to include different statistical-based metrics that could help in the detection process. The current state of the application results in some issues when the window size is large, which implies that the switches stop responding to the requests of states by the control plane. That is why an exhaustive study will be carried out to check if the problem corresponds to the architecture of the switch or to the SDN application. Besides, we will experiment with more complex cases outside Mininet and even make use of other proposals in the stateful SDN literature, experimenting also with entropy-based algorithms on real environments with hundreds of hosts generating network traffic to check the effectiveness of the solution in this context. Moreover, intelligent mechanisms will be introduced, such as Machine Learning techniques that allow self-configuring the algorithm parameters, that is, θ and the window size. Finally, we are working on a (D)DoS attack detector as a Network Function that could be deployed in the network as a standalone module. This would give flexibility to the detection process and would be in line with other 5G technology pillars such as NFV.

## Figures and Tables

**Table 1:** Summary of SDN security threats.

Attack Type	Security Property	SDN NE	SDN Controller	SDN Application
Spoofing	Authentication	vulnerable	vulnerable	vulnerable
Tampering	Integrity	vulnerable	vulnerable	vulnerable
Repudiation	Non-repudiation	-	vulnerable	-
Information disclosure	Confidentiality	vulnerable	vulnerable	vulnerable
Denial of service (DoS)	Availability	vulnerable	vulnerable	vulnerable
Distributed DoS (DDoS)	Availability	vulnerable	vulnerable	vulnerable
Elevation of privileges	Authorization	vulnerable	vulnerable	-

**Table 2:** Certainty that a threat is occurring based on the accuracy of the distribution.

θ	Certainty
1	68%
2	95%
3	99.7%

**Table 3:** Results of experimentation with IoT traffic for different window and θ values.

window - θ	Detection Rate	False Positive Rate	Mean (ms)	Standard Deviation (ms)
**3 - 1**	100%	90%	16.32	2.17
**3 - 2**	100%	20%	20.20	6.38
**3 - 3**	90%	20% *	19.06	9.27
**5 -1**	100%	70%	19.09	8.30
**5 - 2**	100%	70%	20.96	7.04
**5 - 3**	100%	20% *	19.30	3.66
**10 - 1**	100%	60%	22.82	10.38
**10 - 2**	100%	60%	21.41	7.25
**10 - 3**	100%	20% *	21.27	8.56
**20 - 1**	100%	80%	26.93	13.45
**20 - 2**	100%	40%	24.03	14.14
**20 - 3**	80%	20% *	26.98	10.89
